# Irregular anatomical features can alter hemodynamics in Takayasu arteritis

**DOI:** 10.1016/j.jvssci.2023.100125

**Published:** 2023-08-24

**Authors:** Yu Zhu, Xiao Yun Xu, Justin Mason, Saeed Mirsadraee

**Affiliations:** a Department of Chemical Engineering, Imperial College London, London, UK; b Rheumatology and Vascular Science, Hammersmith Hospital, Imperial College London, London, UK; c Department of Radiology, Royal Brompton and Harefield Hospitals, London, UK; d National Heart and Lung Institute, Imperial College London, London, UK

**Keywords:** Takayasu arteritis, Computational fluid dynamics, Hemodynamic parameters, Anatomical features

## Abstract

**Objective:**

Takayasu arteritis (TA) is a difficult disease to deal with because there are neither reliable clinical signs, laboratory biomarkers, nor a single noninvasive imaging technique that can be used for early diagnosis and disease activity monitoring. Knowledge of aortic hemodynamics in TA is lacking. This study aimed to fill this gap by assessing hemodynamics in patients with TA using image-based computational fluid dynamics (CFD) simulations.

**Methods:**

Eleven patients with TA were included in the present study. Patient-specific geometries were reconstructed from either clinical aortic computed tomography angiography or magnetic resonance angiography studies and coupled with physiological boundary conditions for CFD simulations. Key anatomical and hemodynamic parameters were compared with a control group consisting of 18 age- and sex-matched adults without TA who had healthy aortas.

**Results:**

Compared with controls, patients with TA had significantly higher aortic velocities (0.9 m/s [0.7, 1.1 m/s] vs 0.6 m/s [0.5, 0.7 m/s]; *P* = .002), maximum time-averaged wall shear stress (14.2 Pa [9.8, 20.9 Pa] vs 8.0 Pa [6.2, 10.3 Pa]; *P* = .004), and maximum pressure drops between the ascending and descending aorta (36.9 mm Hg [29.0, 49.3 mm Hg] vs 28.5 mm Hg [25.8, 31.5 mm Hg]; *P* = .004). These significant hemodynamic alterations in patients with TA might result from abnormal anatomical features including smaller arch diameter (20.0 mm [13.8, 23.3 mm] vs 25.2 mm [23.3, 26.8 mm]; *P* = .003), supra-aortic branch diameters (21.9 mm [18.5, 24.6 mm] vs 25.7 mm [24.3, 28.3 mm]; *P* = .003) and descending aorta diameter (14.7 mm [12.2, 16.8 mm] vs 22.5 mm [19.8, 24.0 mm]; *P* < .001).

**Conclusions:**

CFD analysis reveals hemodynamic changes in the aorta of patients with TA. The applicability of CFD technique coupled with standard imaging assessments in predicting disease progression of such patients will be explored in future studies. Future large cohort study with outcome correlation is also warranted.

**Clinical Relevance:**

Based on patient-specific computational fluid dynamics simulations, the present retrospective study revealed significant difference in aortic hemodynamics between the patients with and without Takayasu arteritis (TA). To the best of our knowledge, this study is the first to evaluate hemodynamic conditions within TA, demonstrating the potential of computational flow modeling in capturing abnormal hemodynamic forces, such as high wall shear stress, resulted from irregular morphological changes. In the future, assessing the hemodynamic parameters within patients with TA during the prestenotic period, together with longitudinal computational fluid dynamics studies may allow better monitoring and management of TA.

**Article Highlights**

• **Type of Research**: Multicenter retrospective cohort study
• **Key Findings:** Hemodynamic parameters were compared between 11 patients with Takayasu arteritis (TA) and a control group consisting of 18 healthy aortas. Patients with TA presented with abnormal anatomical features, which led to significantly higher aortic velocities, wall shear stress, and pressure drops between the ascending and descending aorta than controls.
• **Take Home Message:** Altered hemodynamic conditions in TA as results of irregular anatomical features can be quantified using imaging-based computational flow analysis. This technique potentially offers the ability to predict disease progression for individual patients, and this should be further explored in the future.

Takayasu arteritis (TA) is a rare, idiopathic, large vessel vasculitis predominately affecting the aorta and its main branches, as well as the pulmonary artery.^1^ The reported annual incidence of TA in Europe ranges from 0.4 to 3.4 cases per million.^2^, ^3^, ^4^, ^5^

Nonspecific constitutional symptoms such as fever, myalgia, and fatigue in the prestenotic phase of TA may cause significant delays in diagnosis of several months or years until after the development of substantial arterial injury.^1^ Inflammatory lesions of TA are characterized by thickening of the arterial walls, which may result in stenoses and/or aneurysms in approximately 90% and 25% of patients, respectively.^1^^,^^6^, ^7^, ^8^, ^9^ The 10-year mortality rate of TA is ≤5%, but can increase to approximately 33% in patients with cerebral and organ ischemia, aortic valve insufficiency, myocardial infraction owing to restricted regional blood flow, or in those with rapidly progressive aortic complications resulting from aneurysmal expansion and aortic rupture.^10^

Noninvasive imaging techniques, such as computed tomography angiography (CTA), high-resolution ultrasound examination, magnetic resonance angiography (MRA), and positron emission tomography have been used for the diagnosis and follow-up of patients with TA. Despite these techniques having shown potential in disease monitoring, each method has distinct and complementary roles, and no single imaging modality can provide all the information required,^10^ which includes an accurate assessment of lesion extent and anatomy, and inflammatory activity in the arterial wall, facilitation of personalized therapy, and the corresponding response to therapy. Moreover, neither blood tests are specific for TA, nor biomarkers can be used as reliable surrogates for disease activity.^11^

It has been recognized that hemodynamic metrics such as blood flow velocities, wall shear stress (WSS), and pressures play an important role in improved diagnosis, risk stratification of aortic diseases, and prediction of late complications, as well as assessment of treatment outcomes.^12^^,^^13^ Computational fluid dynamics (CFD) combined with medical images allows noninvasive analysis of in vivo conditions, and its predictive power and accuracy have been demonstrated.^14^, ^15^, ^16^, ^17^ For example, the pressure and velocity magnitudes predicted by CFD showed good agreements with clinical measurements, which are important for diagnosis of congenital heart diseases.^14^^,^^15^ Even with more complicated geometric models of aortic dissection, CFD predicted velocities showed good consistency with those measured from four-dimensional flow MR imaging,^16^ and its ability to predict false lumen thrombus formation has also been validated.^17^ However, knowledge of vascular hemodynamic conditions in TA is lacking. Therefore, we aimed to address this gap by detailed analysis of blood flow in patients with TA using image-based CFD simulations. We hypothesized that the results obtained from this study would provide valuable information on vascular status in subjects with TA, which can potentially improve disease monitoring and management.

## Methods

### Study design and cohort

Thirteen patients diagnosed with TA between 2015 and 2022 at the Imperial College Healthcare NHS trust were retrospectively identified and included in this study. The inclusion criteria included the patients with TA mainly in the aorta, and with either CTA or MRA images. From the 13 patients, 2 were excluded from CFD simulations owing to inadequate image quality; hence, a total of 11 patients were included in the final analysis. Additionally, 18 age- and sex-matched patients who underwent CTA between 2020 and 2021 at the Royal Brompton and Harefield hospitals for minimal access mitral valve surgery were included as controls. The imaging data of controls were examined by a radiologist with >10 years of experience to ensure all the aortas are without significant tortuosity and clinical signs of dilatation, atherosclerotic, and any other diseases. Patient-specific geometries were reconstructed from either CTA or MRA images, which were then coupled with physiologically realistic boundary conditions. Key geometric features together with hemodynamic parameters, including blood flow velocities, time-averaged WSS (TAWSS), and pressures were analyzed and compared between the two groups.

This study involved human participants, and all procedures were performed in accordance with the ethical standards of the institutional and/or national research committee and with the 1964 Helsinki declaration and its later amendments or comparable ethical standards. The use of patients’ data with TA was approved by Imperial College Healthcare, while the controls were approved by the Institutional committee of Health Research Authority and Health and Care Research Wales. The need for informed consent was waived for this retrospective study.

### Aortic imaging and geometry reconstructions

MRA in 4 of 11 patients with TA were performed on a 1.5 T MRI Scanner (MAGNETOM Aera, Siemens Healthineers, Erlangen, Germany) with a mean slice thickness and increment being 2.0 mm and 1.6 mm, respectively. CTA was performed for the other seven patients using a 128-slice CT scanner (SOMATOM Definition Flash, Siemens Healthineers) and the images were reconstructed with 2.1-mm slice thickness and 1.7-mm slice increment on average. Regarding the control group, all the patients underwent CTA examinations using a 256-slice multidetector CT scanner (Revolution, GE Healthcare, Chicago, IL), where the slice thickness and increment of all CTA images were 0.625 mm. It should be mentioned that both MRA and CTA studies were included for the TA group to increase its sample size. Although the CTA and MRA images used to reconstruct the Takayasu aortas had lower resolutions compared with the control group, they were sufficient to include all the important geometric features.

**Fig 1:**
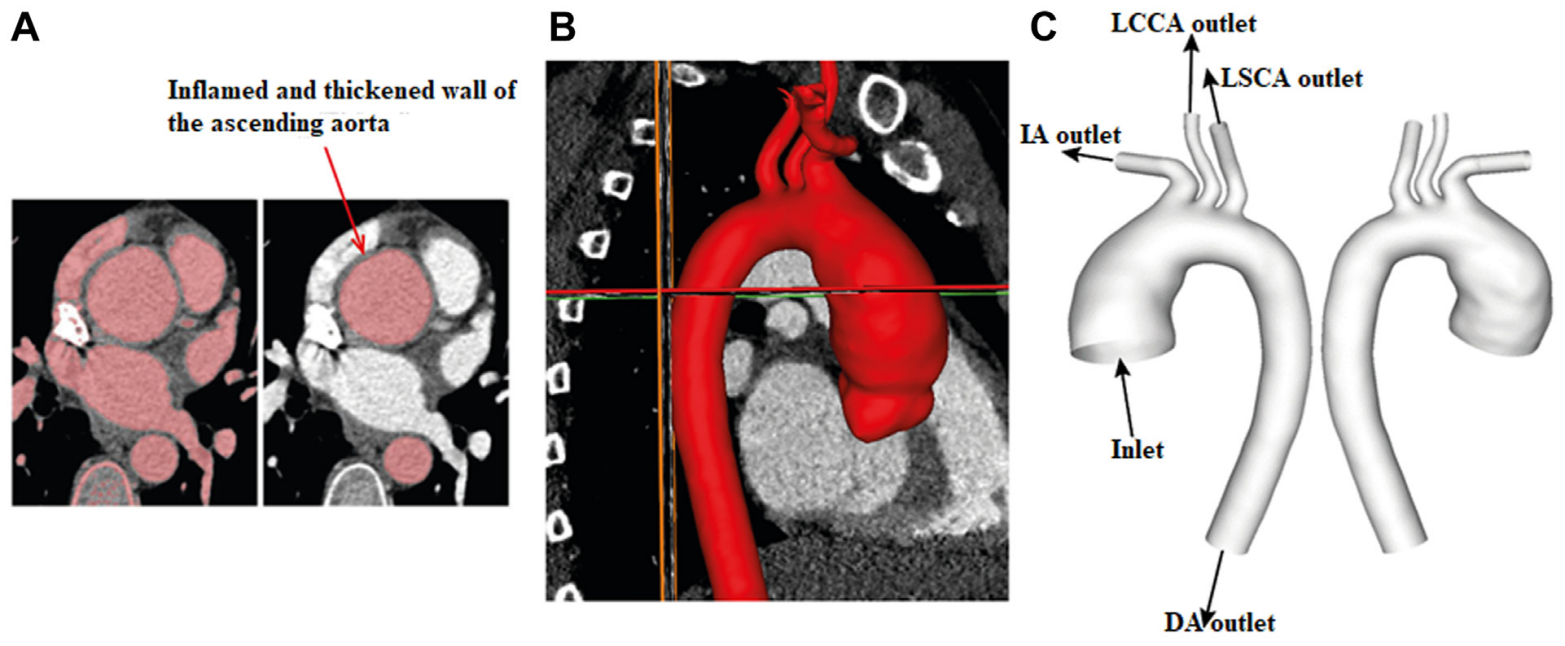
Schematic steps of three-dimensional (3D) geometry reconstruction. **(A)** Target lumen areas are identified and segmented from cross-sectional slices. Inflamed and thickened wall of the ascending aorta (AA) are pointed out by a red arrow. **(B)** The 3D geometry is generated by integrating two-dimensional masks, after which the geometry surface is smoothed. **(C)** The fluid domain is created from sinotubular junction to the level of diaphragm with model inlet and outlets being defined. *DA*, descending aorta; *IA*, innominate artery; *LCCA*, left common carotid artery; *LSCA,* left subclavian artery.

Three-dimensional (3D) geometries of the aortas were reconstructed using a semi-automatic threshold-based segmentation tool (Mimics 24.0, Materialise HQ, Leuven, Belgium). The masks containing the regions of interest (aorta) were manually segmented slice by slice based on the local greyscale intensity (Fig 1, A). The segmented two-dimensional masks were then integrated to generate a 3D geometry, which was smoothed to eliminate any reconstruction errors (Fig 1, B). Finally, the computed region was created from the sinotubular junction (inlet) to the level of diaphragm (descending aorta [DA] outlet) with three main arch branches being included (Fig 1, C). All reconstructed geometries can be found in Supplementary Figs 1, *A* and B. An intraoperator reproducibility study was carried out with six geometries (2 healthy aortas from control group and 4 Takayasu aortas) being reconstructed twice by the same operator (Y.Z.), and the maximum difference in aortic diameter measurements between the original and repeated reconstructions was 0.8 mm. The detailed results are summarized in Supplementary Table I.

After geometry reconstruction, morphological measurements were taken using Mimics software including best-fit diameters and tortuosity, where tortuosity was defined as the ratio between the distance along the centerline and the linear distance. Using ANSYS ICEM CFD (ANSYS, Canonsburg, PA), all the 3D geometries were discretized into fine computational meshes, which consisted of tetrahedral elements in the core and 10 layers of prismatic cells near the wall. Grid independent tests were conducted, and the number of elements adopted in the final analysis for patients with TA and controls ranged from 1.1 to 1.8 million, and from 1.1 to 2.5 million, respectively. The final adopted mesh was considered sufficient since differences in maximum WSS and velocity between the adopted mesh and a finer mesh were <5% and <3%, respectively.

### Blood flow simulations

**Fig 2:**
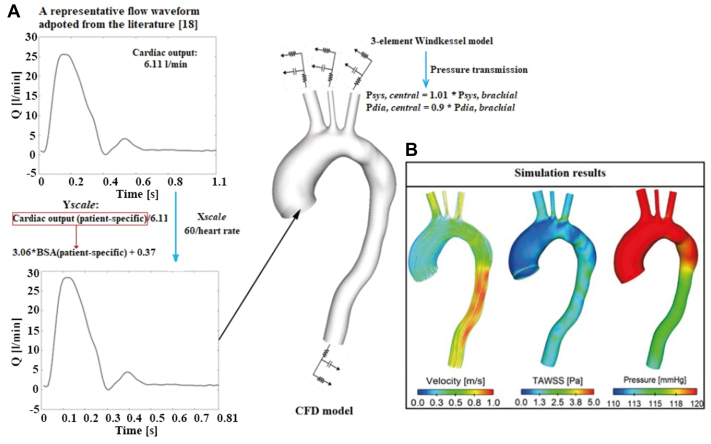
Schematic of the computational model employed in this study. **(A)** A representative flow waveform adopted from the literature^18^ was scaled using patient-specific heart rate and cardiac output calculated from the body surface area (*BSA*). This flow waveform was then prescribed at the model inlet along with the assumption of a flat velocity profile, while a three-element Windkessel model tuned from brachial pressure measurements was applied at all outlets. **(B)** Simulation results including flow patterns, time-averaged wall shear stress (*TAWSS*) and pressures were analyzed.

Physiological boundary conditions were applied to generate clinically relevant results. Briefly, a representative aortic flow waveform at the sinotubular junction was obtained from the literature,^18^ and its amplitude (the peak systolic flow rate) was scaled based on the patient-specific cardiac output calculated from patients’ body surface area (m^2^).^19^ The period of the cardiac cycle was also scaled based on the patients’ heart rate. This scaled flow waveform was applied at the model inlet (Fig 2, A). Patient-specific brachial pressures were converted into central blood pressures,^20^ which were used to tune parameters in the three-element Windkessel model applied at the model outlets. The aortic wall was assumed to be rigid. It should be mentioned that patients’ physiological data (eg, brachial pressure and heart rate) were obtained from the echo reports, and the report with a date close to the CTA or MRA imaging date was used if multiple measurements were performed. Moreover, as shown in Table I, the pressure and heart rate values varied among the patients and groups, and their effects on the predicted results were investigated. The detailed methods and the corresponding results can be found in the Supplementary Material S3.

**Table I:** Patients' characteristics, geometric, and hemodynamic parameters are compared between patients with Takayasu arteritis (TA) and controls

Characteristics	Patients with TA (n = 11)	Controls (n = 18)	*P* value
Female, n (%)	10 (90.9)	16 (88.9)	N/A
Age at examination, years	38.3 ± 9.8	40.5 ± 9.4	N/A
BSA, m^2^	1.9 [1.8 to 2.0]	1.9 [1.7 to 2.0]	.842
Systolic pressure, mm Hg	118.0 [115.0 to 150.0]	131.0 [120.0 to 137.8]	.740
Diastolic pressure, mm Hg	68.0 [60.0 to 78.0]	68.0 [59.8 to 78.0]	.808
Heart rate	64.0 [60.0 to 75.0]	72.5 [68.3 to 81.3]	.188
Geometric parameters			
Maximum aortic diameter, mm	31.6 [30.1 to 38.3]	32.5 [29.6 to 36.4]	.982
Model inlet diameter (D1), mm	28.5 [25.7 to 38.3]	31.4 [26.9 to 32.6]	.492
Arch diameter (D2), mm^a^	20.0 [13.8 to 23.3]	25.2 [23.3 to 26.8]	.003
Arch vessels diameters			
IA outlet diameter, mm	8.7 [7.1 to 10.1]	11.3 [10.1 to 12.2]	.007
LCCA outlet diameter, mm	5.8 [5.3 to 7.9]	6.5 [6.0 to 7.0]	.387
LSCA outlet diameter, mm	6.8 [5.5 to 7.9]	8.8 [7.5 to 10.1]	.004
Arch vessels diameters in total, mm	21.9 [18.5 to 24.6]	25.7 [24.3 to 28.3]	.003
DA outlet diameter (D3), mm	14.7 [12.2 to 16.8]	22.5 [19.8 to 24.0]	<.001
Diameter ratio			
D1/D2	1.6 [1.4 to 1.9]	1.2 [1.1 to 1.3]	.001
D1/D3	2.1 [1.8 to 2.3]	1.3 [1.3 to 1.5]	<.001
Tortuosity	1.9 [1.8 to 2.3]	1.6 [1.6 to 1.7]	<.001
Hemodynamic parameters			
Maximum velocity, m/s	0.9 [0.7 to 1.1]	0.6 [0.5 to 0.7]	.002
Maximum TAWSS, Pa	14.2 [9.8 to 20.9]	8.0 [6.3 to 10.6]	.004
Spatial mean TAWSS, Pa	2.3 [1.7 to 3.4]	1.2 [1.1 to 1.4]	<.001
Maximum PD from inlet to DA outlet, mm Hg	36.9 [29.0 to 49.3]	28.5 [25.8 to 31.5]	.004
Minimum PD from inlet to DA outlet, mm Hg	−12.4 [–16.1 to −10.1]	−12.2 [–14.7 to −11.0]	.774

*BSA*, Body surface area; *DA*, descending aorta; *IA*, innominate artery; LCCA*,* left common carotid artery; *LSCA*, left subclavian artery; *PD*, pressure drop.

Values are number (%), mean ± standard deviation, or median [25 percentile, 75 percentiles].

a Arch diameter was measured 2 cm distal to the origin of the LSCA.

Blood flow through these patient-specific aorta models were simulated by solving the Navier-Stokes equations in conjunction with the correlation-based shear stress transport transitional model^21^ using ANSYS CFX 19.2 (ANSYS, Canonsburg, PA). Blood was assumed to be incompressible and Newtonian with a constant density of 1060 kg/m^3^ and dynamic viscosity of 4 mPa/s. All simulations were performed for five cardiac cycles to achieve a periodic solution, and results including flow patterns, WSS, and pressures obtained in the last cycle were used for detailed analysis (Fig 2, B). All simulations were performed on cluster using 16 cores, with an approximate computational time of 30 to 54 hours per simulation.

### Statistical analyses

All statistical analyses were carried out using SSPS v. 23.0 (IBM Corp., Armonk, NY). Given the small number of patients, comparisons of anatomical and hemodynamic parameters between patients with TA and the controls were performed using the nonparametric Mann-Whitney *U* test, and the results were reported as interquartile range with median [25 percentile, 75 percentiles]. In addition, Spearman’s correlation was used to determine associations between anatomical and hemodynamic parameters. A *P* value of <.05 was considered statistically significant.

## Results

### Anatomical features

This study included 11 patients with TA and 18 normal aortas as controls. Key characteristics and geometric parameters were compared between the two groups of patients, and the results are summarized in Table I. Ten of 11 patients with TA are female (90.9%) with a mean age at examination of 38.3 ± 9.8 years, compared with the control group consisting of 16 female patients (88.9%) and 2 male patients with a mean age of 40.5 ± 9.4 years.

Detailed demographic data of patients with TA, including diagnosis, state of the disease, received treatment at the time of CT/MR imaging, and so on. are summarized in Table II. The mean age at diagnosis was 30.5 ± 3.5 years based on available data in 6 of 11 patients. According to the imaging analysis of inflammation, we found six patients with TA had active disease and the other five patients had inactive disease at the time of scan. Despite the difference in disease activity, all patients with TA developed complications with aortic stenoses being observed in eight patients and aneurysms in three patients. One patient underwent surgical repair with a stent graft implantation, whereas all the other patients were managed medically.

**Table II:** Detailed demographics and imaging findings of patients with Takayasu arteritis (TA)

Patient # (sex/age)	Age at diagnosis	State of the disease	Imaging findings	Treatment received
1 (F/59 years)	32 years	Inactive	Stenosed arch and descending thoracic aorta, stable after endovascular treatment	Endovascular stent at 46 years, and no active immunosuppressants
2 (F/25 years)	24 years	Inactive	Dilated aortic root and ascending thoracic aorta	No active immunosuppressants
3 (F/29 years)	29 years	Active	Dilated ascending thoracic aorta	Methotrexate and prednisolone
4 (F/45 years)	N/A	Active	Stenosed descending thoracic aorta	Infliximab, prednisolone, methotrexate, and clopidogrel
5 (M/38 years)	N/A	Active	Dilated aortic root and ascending thoracic aorta	No active immunosuppressants
6 (F/42 years)	N/A	Inactive	Stenosed arch and descending thoracic aorta	Methotrexate and prednisolone
7 (F/47 years)	N/A	Inactive	Stenosed arch and descending thoracic aorta	No active immunosuppressants
8 (F/30 years)	N/A	Active	Stenosed arch and descending thoracic aorta	Infliximab, prednisolone, and methotrexate
9 (F/35 years)	33 years	Active	Stenosed descending thoracic aorta	Methotrexate and prednisolone
10 (F/41 years)	33 years	Inactive	Stenosed arch and descending thoracic aorta	No active immunosuppressants
11 (F/33 years)	32 years	Active	Stenosed descending thoracic aorta	Azathioprine and Prednisolone

*F*, Female; *M*, male; *N/A*, not applicable.

Dimensions of the supra-aortic arch vessels (sum of three vessels’ diameters measured at the model outlets: 21.9 mm [18.5, 24.6 mm] vs 25.7 mm [24.3, 28.3 mm]; *P* = .003), the DA diameters (D_DA_) measured at the level of diaphragm (14.7 mm [12.2, 16.8 mm] vs 22.5 mm [19.8, 24.0 mm]; *P* < .001), as well as the aortic arch diameters (D_Arch_) measured 2 cm distal to the origin of the left subclavian artery (LSCA) (20.0 mm [13.8, 23.3 mm] vs 25.2 mm [23.3, 26.8 mm]; *P* = .003) were all significantly lower in patients with TA. Two patients with TA developed ascending aortic dilatation with maximum diameters of 44.8 mm and 45.4 mm, respectively. However, no obvious differences in either maximum aortic diameter or proximal ascending aorta (AA) diameter (D_AA_) measured at the model inlet were found between the two groups. As a result, significantly higher diameter ratios of D_AA_/D_Arch_ (1.6 [1.4, 1.9] vs 1.2 [1.1, 1.3]; *P* = .001), and D_AA_/D_DA_ (2.1 [1.8, 2.3] vs 1.6 [1.6, 1.7]; *P* < .001) were observed in patients with TA. The tortuosity was found to vary from 1.6 to 2.7 in patients with TA, compared with 1.4 to 1.8 in the control group. Patients with TA had a significantly higher degree of tortuosity (1.9 [1.8, 2.3] vs 1.6 [1.6, 1.7]; *P* < .001).

### Flow patterns

**Fig 3:**
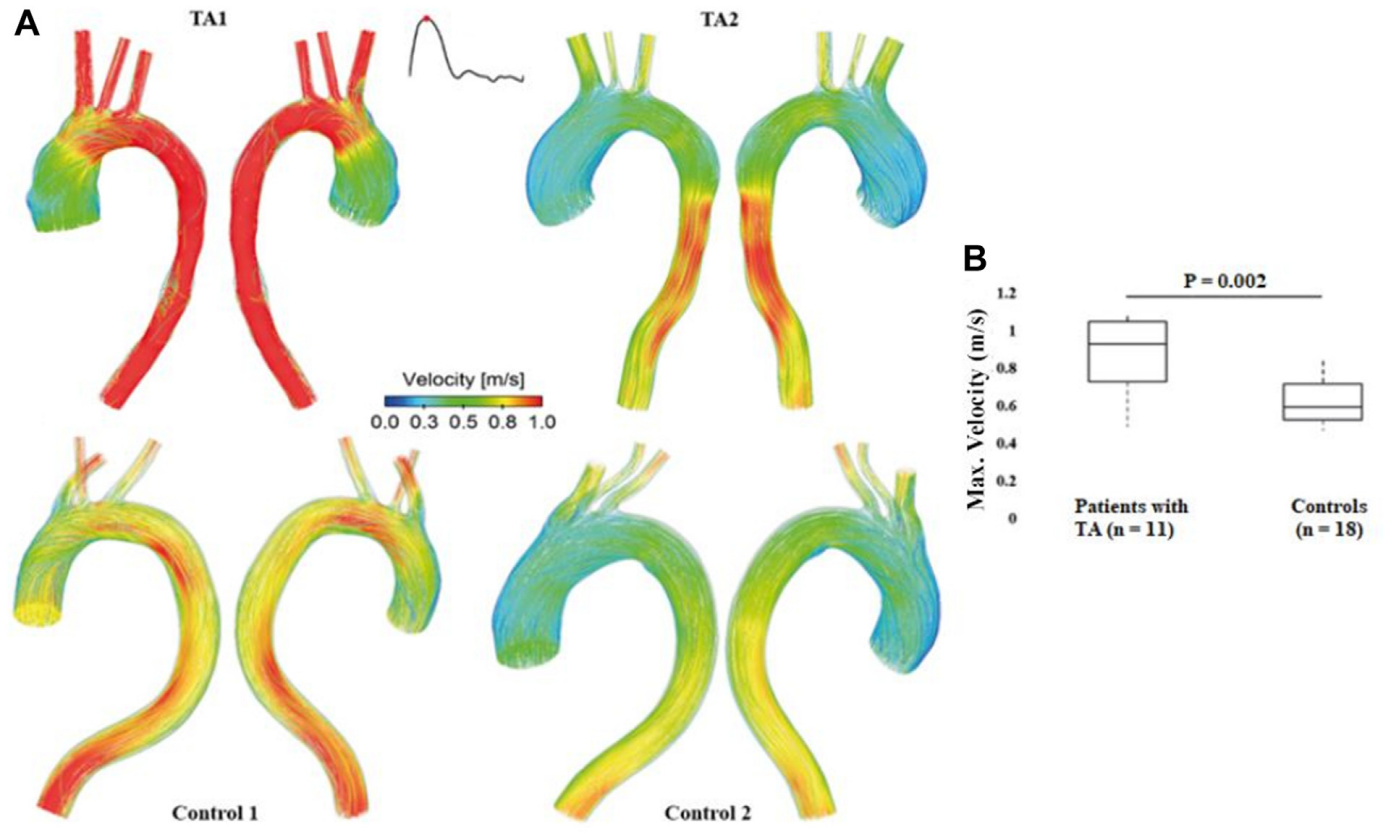
Instantaneous velocity streamlines for four selected cases at peak systole. **(A)** Flow patterns are compared between two patients with Takayasu arteritis (*TA*) (above) and two samples from the control group (below). Velocities >1 m/s are shown in red. Much higher blood flow velocities can be observed in patients with TA. (**B)** Comparisons of the maximum velocities over a cardiac cycle showing patients with TA had significantly higher velocities than controls (*P* = .002).

Instantaneous velocity streamlines at peak systole in four cases (two from each group) are displayed in Fig 3, A. Common flow features can be observed in both groups: blood flow accelerations from the distal AA, or from regions distal to the aortic arch as a result of reduced lumen area; helical flows rising from both inner and outer wall of the AA up to the upper aortic arch; a global left-handed helical pattern in the DA, more obvious in the control group. Despite the overall flow patterns are comparable, much higher blood velocities were observed in patients with TA, especially for TA1, who had significantly narrowed arch (13.7 mm) and DA (12.4 mm) diameters. Flow patterns for all the other patients are shown in (Supplementary Figs 2*A* and *B*).

A comparison of the maximum velocity (normalized as spatial-mean velocity over the entire fluid domain) (Fig 3, B) between the two groups suggested that patients with TA had significantly higher velocities than controls (0.9 m/s [0.7, 1.1 m/s] vs 0.6 m/s [0.5, 0.7 m/s]; *P* = .002). Moreover, velocity was found to strongly correlate with the following geometric features: arch diameter (R = −0.859; *P* < .001), D_DA_ (R = −0.809; *P* < .001), sum of arch vessels diameters (R = −0.769; *P* < .001), and tortuosity (R = 0.631; *P* < .001).

### TAWSS

**Fig 4:**
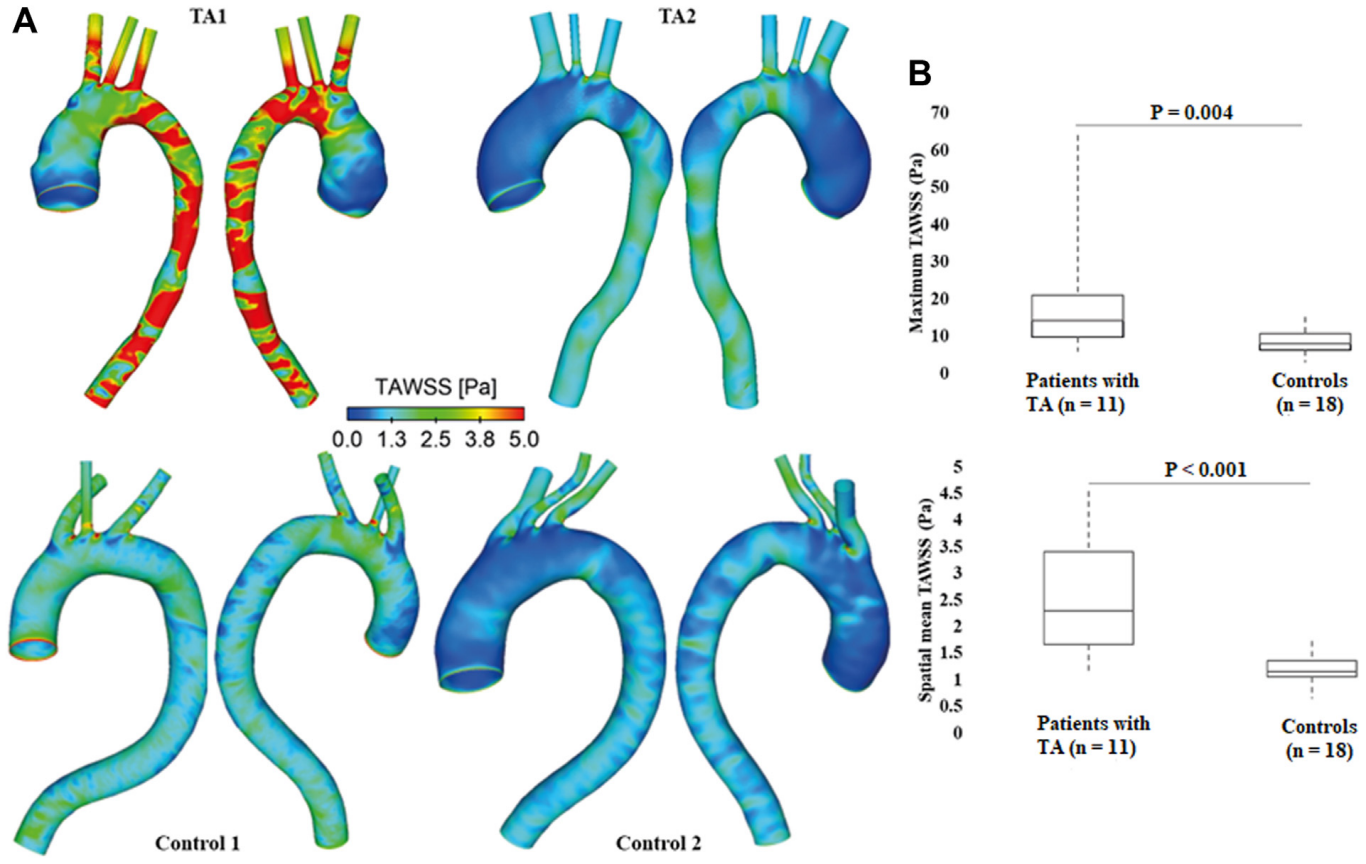
Comparison of time-averaged wall shear stress (*TAWSS*) in selected patients. **(A)** TAWSS distributions are compared between two patients with Takayasu arteritis (*TA*) (above) and two samples from the control group (below). TAWSS magnitudes of >5 Pa are shown in red. Similar to flow patterns, much higher TAWSS can be observed in patients with TA. **(B)** Patients with TA had significantly higher TAWSS magnitudes, as represented by the peak values (*P* = .004) and spatial-averaged values (*P* < .001).

WSS is the frictional force exerted by blood flow on the inner wall, which can be calculated based on velocity gradient at the wall and blood viscosity. The magnitude and direction of WSS change during the cardiac cycle because of the pulsatile nature of blood flow; as such, it is usually analyzed by averaging the magnitude of instantaneous WSS over a cardiac cycle to yield a cycle-averaged value, referred to as time-averaged WSS (TAWSS). The TAWSS contours shown in Fig 4, A demonstrate similar spatial distributions to flow patterns, with shear stress being lower in the AA than in the arch and DA. High TAWSS magnitudes were localized at the junctions of arch vessels, and regions with reduced diameters. TAWSS distributions for all the patients can be found in Supplementary Fig 3, *A* and *B*.

The TAWSS magnitudes were also found to be significantly higher in patients with TA owing to greater blood velocities. Peak TAWSS values varied from 5.9 Pa to 64.2 Pa in patients with TA, compared with 2.9 to 15.1 Pa in controls. Both maximum (14.2 Pa [9.8, 20.9 Pa] vs 8.0 Pa [6.2, 10.3 Pa]; *P* = .004) and spatial-mean TAWSS magnitudes (2.3 Pa [1.7, 3.4 Pa] vs 1.2 Pa [1.1, 1.4 Pa]; *P* < .001) were significantly higher in patients with TA (Fig 4, B). Small diameters of the arch and its vessels, as well as the DA, but high degree of tortuosity, could all be correlated with large TAWSS magnitudes (Table III).

**Table III:** Correlations were made between geometric and hemodynamic parameters (Spearman's test)

Geometric parameters	Hemodynamic parameters			
	Maximum velocity	Maximum TAWSS	Mean TAWSS	Maximum PD
Arch diameters	−0.859^a^	−0.794^a^	−0.842^a^	–0.474^a^
Inlet/arch diameter ratio	0.355	0.345	0.443^b^	0.335
DA outlet diameters	−0.809^a^	−0.801^a^	−0.855^a^	−0.407^b^
Inlet/D_DA_ ratio	0.429^b^	0.434^b^	0.534^a^	0.244
Sum of arch vessels diameters	−0.769^a^	−0.588^a^	−0.737^a^	−0.359
Tortuosity	0.631^a^	0.698^a^	0.746^a^	0.468^b^

*DA*, Descending aorta; *D*_*DA*_, descending aorta diameter; *PD*, pressure drop; *TAWSS*, time-averaged wall shear stress.

a P < .001.

b *P* < .05.

### Pressure drops

**Fig 5:**
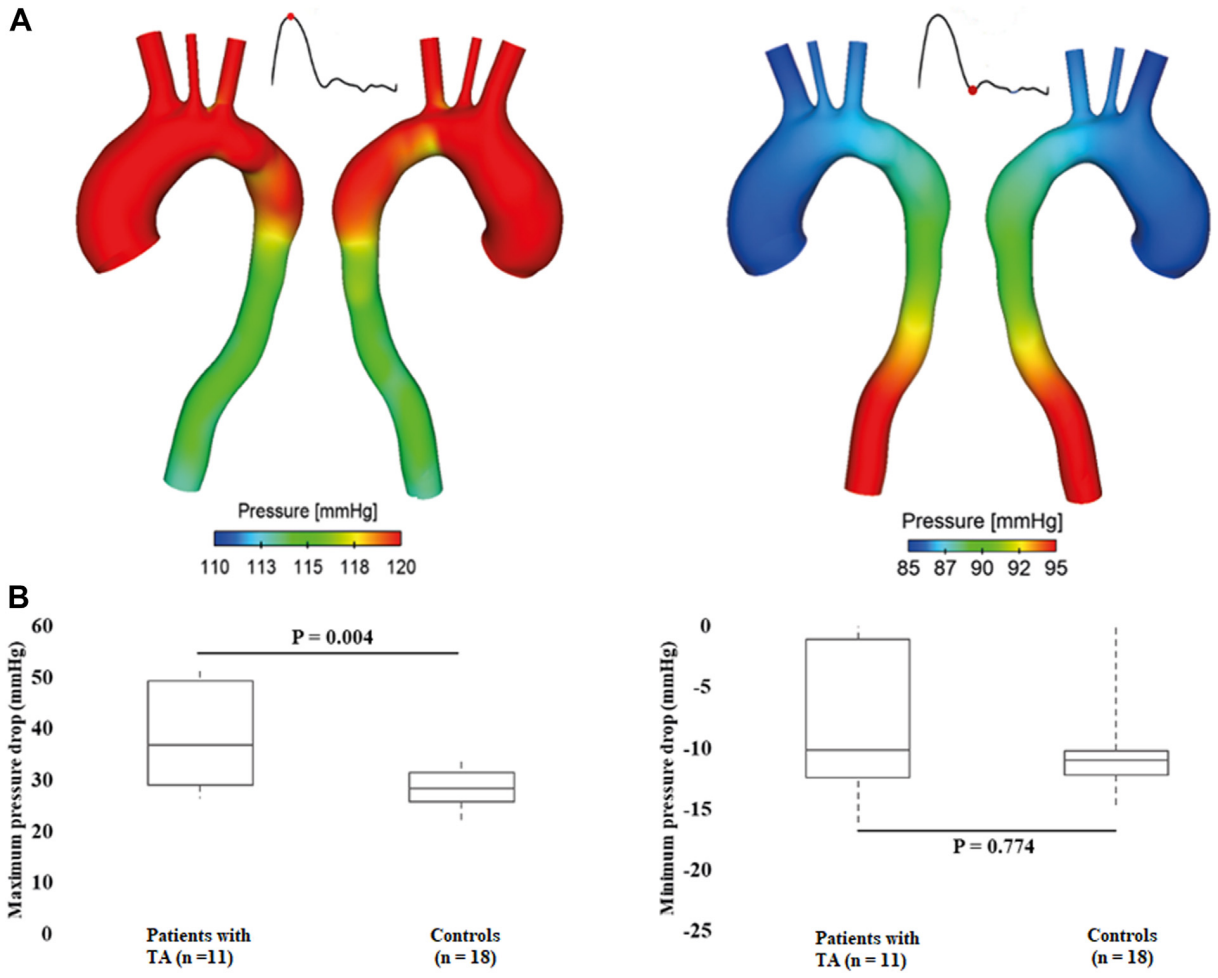
Pressure distributions for one patient with Takayasu arteritis (*TA*). **(A)** Pressure distributions are shown at two characteristic time points, namely, peak systole (left), and early diastole (right). At peak systole, pressure gradually reduced from the ascending aorta (AA) to descending aorta (DA), while the opposite is true during diastole. **(B)** The maximum pressure drops (PD) over a cardiac cycle is significantly higher in patients with TA (*P* = .004), whereas the minimum PD (larger in DA) is comparable between the two groups (*P* = .774).

As shown in Fig 5, A, pressure distributions at two time points were plotted for one representative patient with TA. At peak systole, pressure gradually decreased from the AA to DA, and the opposite is true during diastole. Spatial mean pressures over a cardiac cycle were evaluated for the AA inlet and DA outlet. Then, pressure drops (PDs) from the inlet to DA outlet were evaluated as differences between inlet and DA outlet pressures (PD = P_inlet_ – P_Daoutlet_), and the minimum (negatively maximum) and maximum PD over a cardiac cycle were determined and compared. Only the maximum PD was significantly different between the two groups, with patients with TA presented higher PD values (36.9 mm Hg [29.0, 49.3 mm Hg] vs 28.5 mm Hg [25.8, 31.5 mm Hg]; *P* = .004) (Fig 5, B). Moreover, the maximum PD was found to be moderately correlated with arch (R = −0.474; *P* = .009) and DA (R = −0.407; *P* = .029) diameters, as well as the tortuosity of the aorta (R = 0.468; *P* = .01).

## Discussion

Comparisons of key anatomical and hemodynamic parameters between 11 patients with TA and a control group consisting of 18 healthy aortas showed significantly higher aortic velocities, TAWSS, and PDs between the ascending and DA in patients with TA than controls, which might result from abnormal anatomical features in patients with TA including smaller arch, supra-aortic branch, and D_DA_s.

TA can lead to aortic complications such as stenosis and aneurysm in approximately 90% and 25% of the patients, respectively,^1^^,^^6^, ^7^, ^8^, ^9^ both of which are associated with high mortality rates.^10^ Predicting disease progression in patients with TA is challenging. There are no reliable parameters that can reflect disease activity and the risk of aortic stenosis or occlusion. An accurate assessment of disease activity in TA is crucial for treatment planning, which may be accomplished by the integrated use of clinical symptoms and signs, laboratory assessment, and noninvasive imaging. However, active inflammation was still observed in four of the nine arterial specimens that were obtained from patients with apparent clinical and laboratory remission.^1^ In contrast, CFD is an indispensable tool for blood flow analysis in the cardiovascular system, but its application on TA models is still lacking. This study is the first to evaluate hemodynamic conditions within TA, which may help clinicians better understand the disease process and stratifies patients at risk.

Helical blood flow patterns were observed in all simulated TA and control models at peak systole (Fig 3). The presence of helical flow is a positive sign since it can help prevent flow recirculation, reduce flow stagnation, and enhance oxygen transport and atherogenic lipid washout.^22^^,^^23^ More organized blood flow can be observed in controls without sudden changes in blood velocities. TA1 and TA2 present with two most common late complications of TA, namely, DA stenosis (minimum D_DA_, 12.4 mm) and AA aneurysm (maximum AA diameter, 44.8 mm), respectively. Either an expanded AA or a narrowed DA of patients with TA resulted in larger diameter ratios of D_AA_/D_Arch_ and D_AA_/D_DA_ as compared with controls. As a result of more tapered aortas, blood velocities are much higher in patients with TA.

We identified two patients with TA (TA6 and TA7, as shown in the Supplemental Materials) with an extremely high peak TAWSS magnitudes of 48.1 and 64.2 Pa, respectively. Both patients presented with severe coarctation of aorta distal to the arch, with the minimum diameters being 9.8 mm in TA6 and only 7.9 mm in TA7. Increased velocities at tight stenosis leading to high local WSS has been reported in previous study.^24^ Abnormal high WSS has been associated with endothelial damage, degenerative lesions of the vessel wall, and subsequent vessel enlargement.^25^, ^26^, ^27^, ^28^ In contrast, very low TAWSS values (nearly 0 Pa) was observed throughout the AA of two patients with TA (TA2 as shown in Fig 4) who had aneurysms. WSS values of <0.4 Pa have been suggested to be thrombogenic and may also lead to aortic dilatation.^29^ The peak TAWSS values of the other nine patients with TA are ≤20.9 Pa. To minimize the impact of outliers (ie, two extremely high TAWSS magnitudes) on statistics, spatial-mean TAWSS averaging the TAWSS values over the entire wall surface was also evaluated and compared. Again, patients with TA had significantly higher TAWSS magnitudes.

PD across a stenotic valve or vessel has been widely used to assess the severity of stenosis, according to which the treatment decisions can be made.^30^^,^^31^ CFD offers a noninvasive alternative method to measure aortic PD. Because >90% of patients with TA present with stenoses,^1^ we assumed that the PD in patients with TA would also be different from controls. As expected, the maximum PD calculated over a cardiac cycle was significantly higher in patients with TA. Unlike what have been reported in previous studies on aortic coarctations,^32^^,^^33^ PD in our study was calculated as the pressure difference between the inlet and DA outlet, because stenosis may occur along the entire length of the aorta in patients with TA.^10^ Moreover, PD was found to be negatively correlated with arch (R = −0.474; *P* = .009) and DA (R = −0.407; *P* = .029) diameters. These findings are reasonable; high flow velocities and increased viscous forces resulted from the narrowed vessels can produce greater PD. The PD increases with an increasing stenosis rate and turbulent flow, and to supply sufficient blood perfusion to systemic circulation within the body, a greater PD will increase the workload of the heart.^34^ In addition, high pressure loss downstream of the stenosis can increase the flow resistance, leading to aortic wall collapse.^35^

Early diagnosis in patients with TA before the occurrence of a critical stenosis or occlusion is crucial, but still challenging. CFD has shown its ability in predicting future occurrence of type A aortic dissection by assessing hemodynamics in predissection models.^36^ Inspired by this study, future studies assessing the hemodynamic parameters within patients with TA during the prestenotic period may also help to identify potential predictors for subsequent complications. Regarding disease activity monitoring, longitudinal CFD studies should be performed in future using multiple follow-up scans so that variable aortic morphological changes over time and their impacts on local hemodynamics can be assessed.^37^ Therefore, we believe that image-based CFD has a great potential to provide valuable information on disease diagnosis and monitoring, and with the advances in computational simulation techniques, personalized intervention planning may be achieved by virtual simulations of treatment procedures.^38^^,^^39^ Once thoroughly validated, the same CFD approach can also be applied to evaluate the hemodynamics in other smaller aortic vessels affected by TA, such as occluded carotid arteries which may cause cerebral ischemia.^40^

### Limitations of the study

The wider applicability of the findings in the present study is limited by the small sample size. Nevertheless, the number of patients with TA included in this study is limited considering the annual incidence per million individuals is only 0.8 in the UK.^2^ Any new identified patients will be included in our future studies. Second, the control group consists of aortas that are in normal condition. It may be of particular interest to study other patients with large vessel vasculitis, such as giant cell arteritis; the vascular phenotype between the two groups of patients may be overlapping and traditional discrimination based on the age at disease onset can be inaccurate.^41^ In addition, the potential use of CFD simulation for risk prediction in aortic branch vessels^40^ or pulmonary artery^42^ affected by TA will be explored.

With regard to the computational model, the rigid wall assumption may miss the opportunity to capture some complex flow structures caused by expansion and contraction of the vessel wall.^43^ However, accounting for aortic wall compliance by means of fluid-structure interaction simulations would increase the computational time by 10-fold, and hence would be unsuitable for the present comparative studies. Nevertheless, increased arterial stiffness has been reported for the patients with TA^44^ which may weaken the impact of rigid wall assumption. Moreover, the present study neglects the inflammatory activity within the aortic wall, which can be incorporated with a growth and remodeling model in future studies. Although growth and remodeling models have been widely applied to predict aneurysmal growth,^45^^,^^46^ very few studies have been reported to account for inflammatory cells.^47^^,^^48^ In addition, patient-specific flow data were not available owing to retrospective nature of the study, so that a representative flow waveform obtained from a healthy individual^18^ was scaled based on patient-specific cardiac output. Despite these individually scaled flow waveforms contained some patient-specific features, patient-specific velocity profiles should be used in future if they are available. Moreover, the CTA and MRA images from the TA group had lower axial resolution with a larger slice thickness and increment compared with the control group. Nevertheless, the in-plane resolutions represented by pixel size were comparable: 0.54 to 0.87 mm for the control group, 0.59 to 0.78 mm for the patients with TA with CTA images, and 0.93 mm to 0.97 mm for the patients with TA with MRA images. Relative low resolution of the MRA images for four patients with TA might introduce some errors in the measurement of aortic diameter, which would further result in uncertainties in the predicted WSS values. Therefore, it would be desirable to use high-resolution CTA images in future studies.

## Conclusions

Hemodynamic conditions in 11 patients with TA were assessed by performing patient-specific CFD simulations. Patients with TA presented significantly higher blood flow velocities, TAWSS magnitudes, and PDs from the AA to the DA, as compared with a group of control patients who did not show any aortic diseases. The significant hemodynamic alterations in patients with TA might result from abnormal geometric features including AA aneurysms and DA stenoses. This preliminary pilot study is small, and thus no strong inferences can be drawn; however, with future large cohort studies, CFD may provide valuable information on diagnosis and monitoring of TA.

## Author Contributions

Conception and design: YZ, XX, JM, SM

Analysis and interpretation: YZ

Data collection: YZ, JM, SM

Writing the article: YZ

Critical revision of the article: YZ, XX, JM, SM

Final approval of the article: YZ, XX, JM, SM

Statistical analysis: YZ

Obtained funding: Not applicable

Overall responsibility: SM

## Disclosures

None.
