# Supplementary material for: Irregular anatomical features can alter hemodynamics in Takayasu arteritis
Source: JVS Vasc Sci. 2023 Aug 24;4:100125. doi: 10.1016/j.jvssci.2023.100125 (PMC10522970; doi:10.1016/j.jvssci.2023.100125)
Supplement: Supplementary material [file mmc1.docx]

Supplementary Materials

Irregular anatomical features can alter hemodynamics in Takayasu arteritis

Yu Zhu^a^, Xiao Yun Xu^a^, Justin Mason^b^, Saeed Mirsadraee^c,d^

^a^Department of Chemical Engineering, Imperial College London, London, UK

^b^Rheumatology and Vascular Science, Hammersmith Hospital, Imperial College London, London, UK

^c^Department of Radiology, Royal Brompton and Harefield Hospitals, London, UK

^d^National Heart and Lung Institute, Imperial College London, London, UK

# **Corresponding and the post-publication corresponding author:**

Dr Saeed Mirsadraee, Department of Radiology, Royal Brompton Hospital, Sydney St, Chelsea, London SW3 6NP, United Kingdom Tel: +44(0)207 352 8121 Email: [s.mirsadraee@rbht.nhs.uk](mailto:s.mirsadraee@rbht.nhs.uk)

**Key words**

Takayasu arteritis; computational fluid dynamics; hemodynamic parameters; anatomical features.

**Conflict of interest**

All authors have no conflict of interest to declare.

**Funding statement**

No specific funding was received from any bodies in the public, commercial or not-for-profit sectors to carry out the work described in this article.

# **S1. Geometry reconstructions**


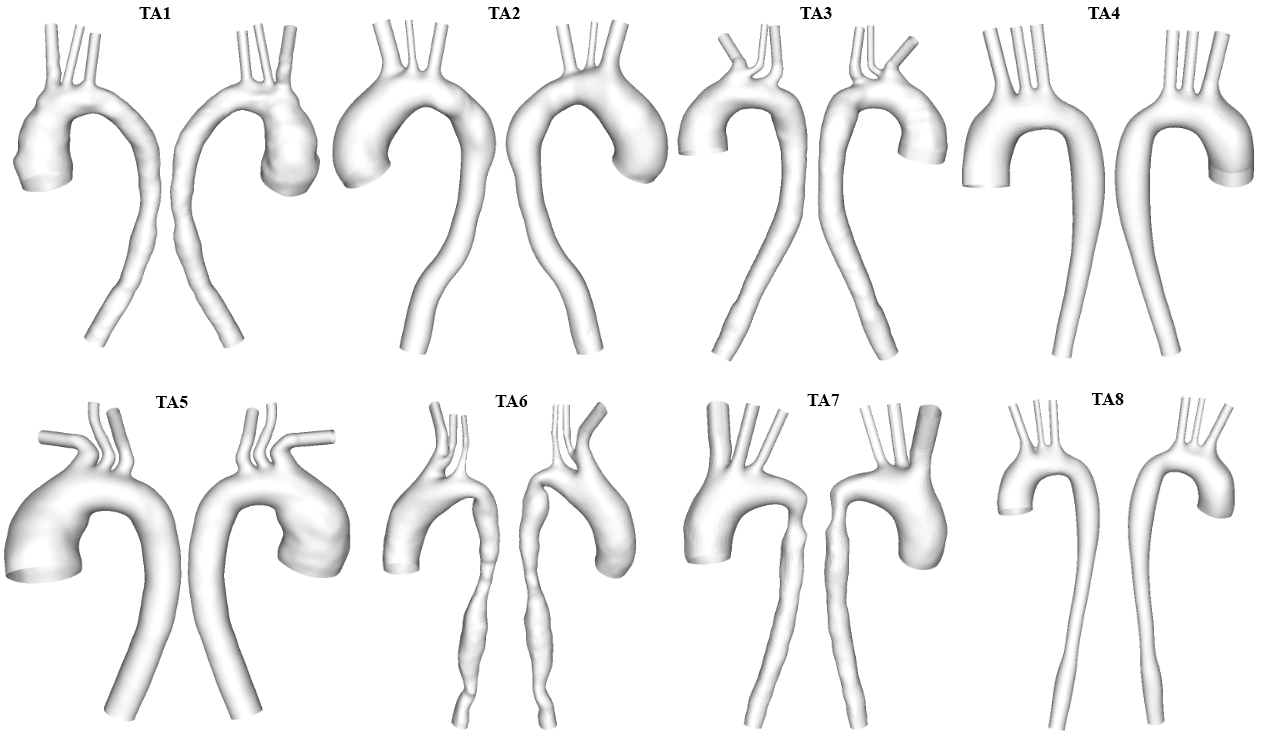


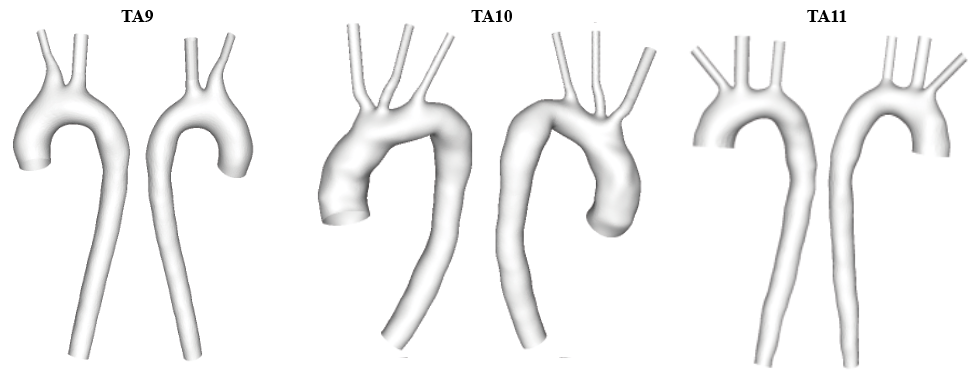


**Figure S1a**. Geometry reconstructions for all patients with Takayasu arteritis (TA).


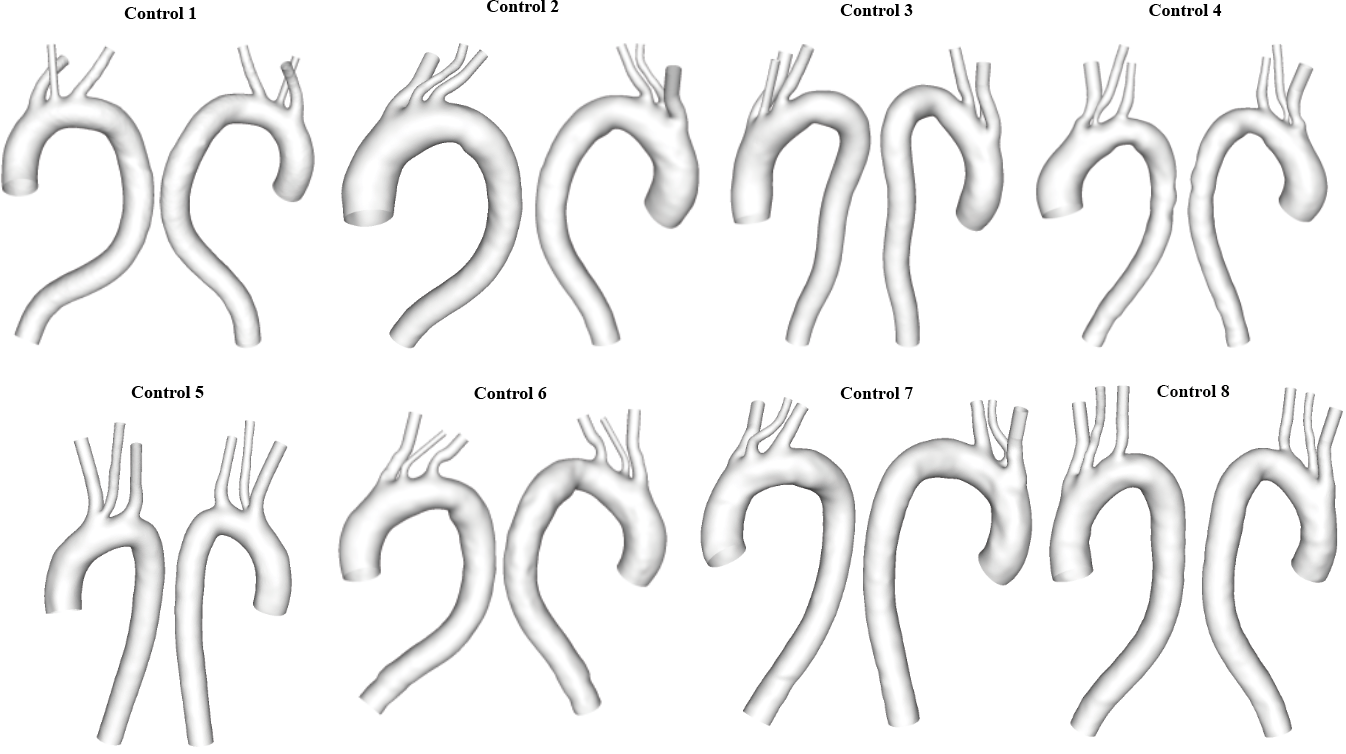


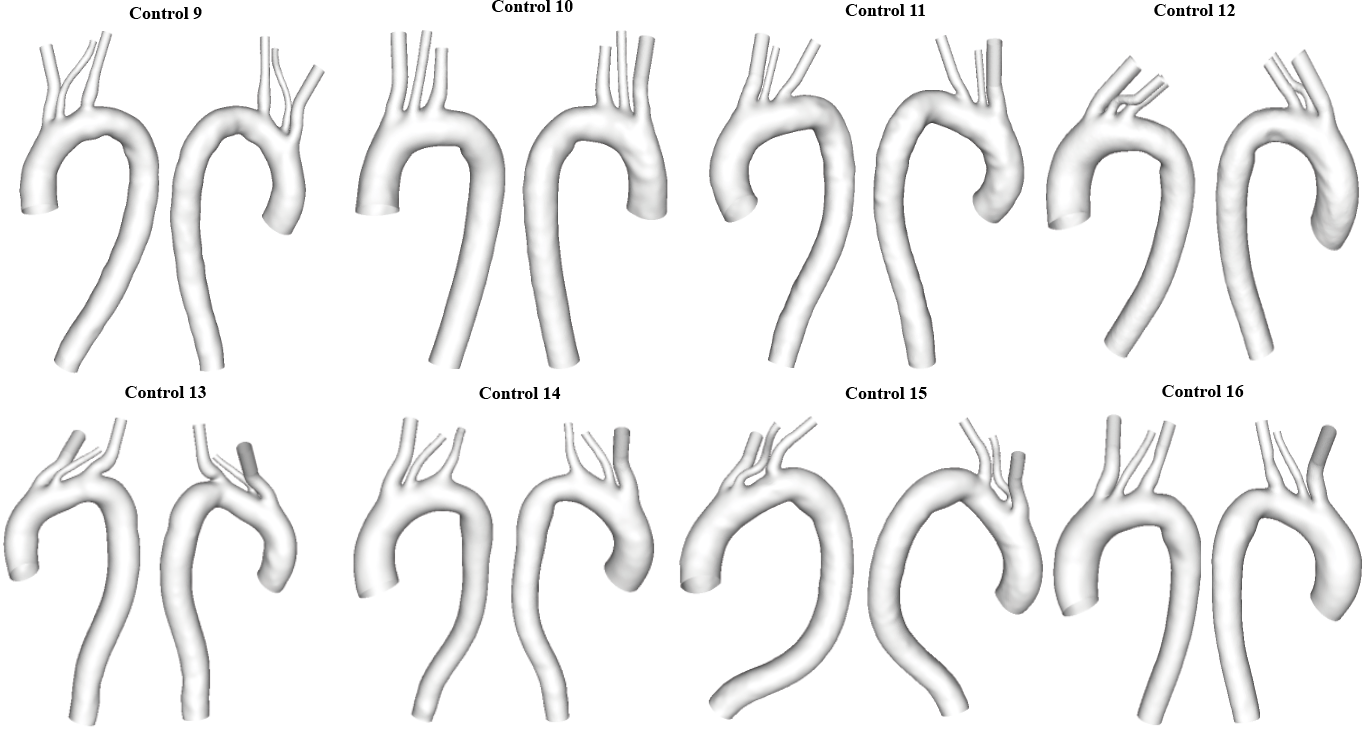


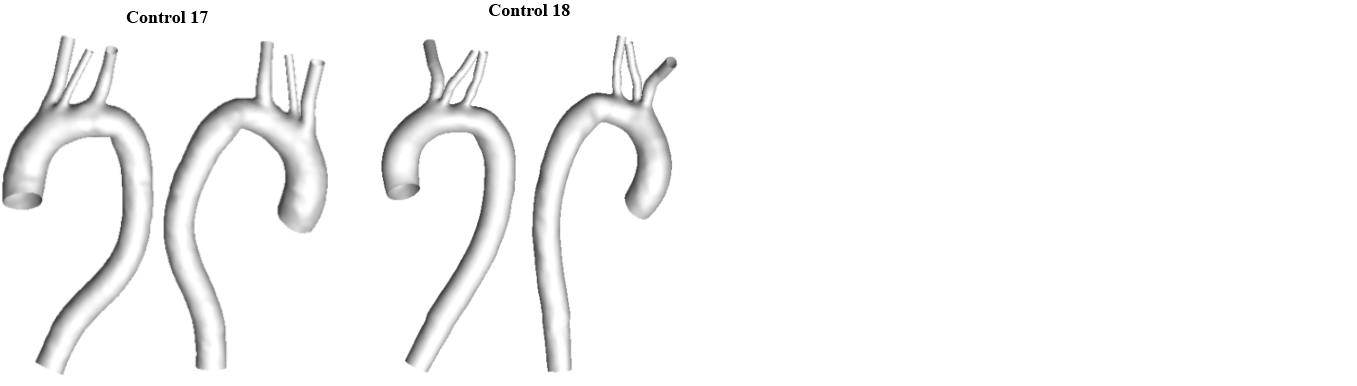


**Figure S1b**. Geometry reconstructions for all patients in the control group.

# **S2. Intra-operator Reproducibility Study**

An intra-operator reproducibility study was carried out to analyse how repeatable the geometry reconstructions in this study were. Six geometries (2 healthy aortas from control group, 4 Takayasu aortas) were reconstructed again by the same operator, without referencing the original reconstructions used in the CFD simulations. Aortic diameters among different locations measured and compared between the original and repeated reconstructions. The results are summarised in Table S1.

**Table S1.** Comparison of aortic diameters between the original and repeated reconstructions.

| **Control Group (CTA Data)** | | | | | | |
| --- | --- | --- | --- | --- | --- | --- |
|  | **Control 6** | | | **Control 10** | | |
|  | **Simulated Model** | **Repeated Model** | **Difference (%)** | **Simulated Model** | **Repeated Model** | **Difference (%)** |
| ***Aortic Diameters*** |  | | |  | | |
| Model inlet diameter (mm) | 31.3 | 32.1 | 2.6 | 27.9 | 27.7 | -0.1 |
| IA outlet diameter (mm) | 10.6 | 11.0 | 3.8 | 10.4 | 10.6 | 1.9 |
| LCCA outlet diameter (mm) | 5.6 | 5.8 | 3.6 | 6.5 | 6.5 | 0 |
| LSCA outlet diameter (mm) | 10.2 | 10.5 | 2.9 | 8.1 | 8.1 | 0 |
| DA outlet diameter (mm) | 24.1 | 24.8 | 2.9 | 22.1 | 22.1 | 0 |
| ***Cross-section Planes (mm)*** |  | | |  | | |
| P1 | 27.4 | 27.7 | 1.1 | 23.7 | 23.6 | -0.4 |
| P2 | 31.3 | 31.7 | 1.3 | 24.7 | 24.5 | -0.8 |
| P3 | 28.6 | 29.1 | 1.7 | 23.7 | 23.5 | -0.8 |
| P4 | 27.7 | 28.3 | 2.2 | 22.9 | 22.4 | -2.2 |
| P5 | 26.6 | 27.2 | 2.3 | 22.2 | 22.0 | -0.9 |
| P6 | 26.6 | 27.1 | 1.9 | 22.0 | 21.9 | -0.5 |
| P7 | 26.1 | 26.7 | 2.3 | 21.8 | 21.6 | -0.9 |
| P8 | 24.4 | 25.2 | 3.3 | 22.0 | 21.9 | -0.5 |
| **Takayasu Aorta (CTA Data)** | | | | | | |
|  | **TA2** | | | **TA5** | | |
|  | **Simulated Model** | **Repeated Model** | **Difference (%)** | **Simulated Model** | **Repeated Model** | **Difference (%)** |
| ***Aortic Diameters*** |  |  |  |  |  |  |
| Model inlet diameter (mm) | 40.8 | 40.0 | -2.0 | 42.4 | 43.1 | 1.7 |
| IA outlet diameter (mm) | 13.5 | 13.4 | -0.7 | 10.1 | 10.3 | 2.0 |
| LCCA outlet diameter (mm) | 5.9 | 6.1 | 3.4 | 6.6 | 6.9 | 4.5 |
| LSCA outlet diameter (mm) | 10.3 | 10.7 | 3.9 | 7.9 | 8.2 | 3.8 |
| DA outlet diameter (mm) | 22.8 | 22.5 | 1.3 | 20.8 | 21.3 | 2.4 |
| ***Cross-section Planes (mm)*** |  | | | | | |
| P1 | 26.7 | 25.7 | -3.7 | 23.9 | 24.5 | 2.5 |
| P2 | 30.0 | 29.0 | -3.3 | 23.2 | 23.8 | 2.6 |
| P3 | 23.3 | 22.8 | -2.1 | 22.6 | 23.1 | 2.2 |
| P4 | 21.2 | 20.6 | -2.8 | 22.2 | 22.7 | 2.3 |
| P5 | 21.0 | 20.2 | -3.8 | 21.3 | 21.9 | 2.8 |
| P6 | 21.5 | 20.9 | -2.8 | 20.9 | 21.3 | 1.9 |
| P7 | 23.2 | 22.6 | -2.6 | 21.2 | 21.7 | 2.3 |
| P8 | 23.2 | 22.5 | -3.0 | 20.9 | 21.4 | 2.4 |
| **Takayasu Aorta (MRA Data)** | | | | | | |
|  | **TA6** | | | **TA7** | | |
|  |  |  |  |  |  |  |
| ***Aortic Diameters*** |  |  |  |  |  |  |
| Model inlet diameter (mm) | 26.5 | 27.0 | 1.9 | 25.7 | 25.6 | -0.4 |
| IA outlet diameter (mm) | 9.4 | 9.2 | -2.1 | 13.6 | 13.6 | 0 |
| LCCA outlet diameter (mm) | 5.3 | 5.5 | 3.6 | 7.9 | 7.4 | -6.3 |
| LSCA outlet diameter (mm) | 4.1 | 4.4 | 7.3 | 5.7 | 5.3 | -7.0 |
| DA outlet diameter (mm) | 14.7 | 14.4 | 2.0 | 10.9 | 10.4 | -4.6 |
| ***Cross-section Planes (mm)*** |  |  |  |  |  |  |
| P1 | 13.8 | 13.6 | -1.4 | 16.0 | 16.2 | 1.3 |
| P2 | 15.0 | 14.7 | -2.0 | 8.0 | 8.4 | 5.0 |
| P3 | 10.7 | 10.4 | -2.8 | 14.6 | 15.0 | 2.7 |
| P4 | 11.3 | 11.4 | 0.9 | 11.7 | 12.3 | 5.1 |
| P5 | 19.4 | 20.0 | 3.1 | 13.1 | 13.0 | -0.8 |
| P6 | 17.0 | 16.8 | -1.2 | 12.8 | 13.0 | 1.6 |
| P7 | 10.0 | 10.3 | 3.0 | 11.9 | 12.2 | 2.5 |
| P8 | 13.5 | 13.1 | 3.0 | 11.7 | 12.0 | 2.6 |

# **S3. Evaluation of the impacts of inter- and intra-group variations of patient-specific measurements of pressure and heart rate**

The patient-specific inlet and outlet boundary conditions (BCs) were calculated based on patients’ heart rate (HR) and body surface area (BSA), and patients-specific brachial pressures, respectively. Considering these physiological measurements could fluctuate among the patients and groups, their effects on the predicted results were assessed. By doing so, two different sets of BCs were specified at the outlets of two models (one from each group), whereas the unscaled flow waveform, obtained from [18], was directly applied at the two models’ inlets, shown in Table S2.

**Table S2.** Comparison of different sets of boundary conditions that were applied.

|  | Inlet BC | AoR BC | DAo BC |
| --- | --- | --- | --- |
| **Original BC** | **Scaled flow waveform** | **3-EWM: calibrated using patients’ brachial pressures** | |
| Patient with TA | Mean cardiac output:  6.65 l/min; HR: 67 | Systolic pressure: 167 mmHg  Diastolic pressure: 72 mmHg | |
| Control | Mean cardiac output:  6.15 l/min; HR: 72 | Systolic pressure: 132 mmHg  Diastolic pressure: 58 mmHg | |
| **BC1** | **Unscaled flow waveform** | **3-EWM: calibrated using normal pressures** | |
| Patient with TA | Mean cardiac output:  6.11 l/min; HR: 60 | Systolic pressure: 120 mmHg  Diastolic pressure: 80 mmHg | |
| Control | Mean cardiac output:  6.11 l/min; HR: 60 | Systolic pressure: 120 mmHg  Diastolic pressure: 80 mmHg | |
| **BC2** | **Unscaled flow waveform** | **Mass flow rate (flow split based on outlet areas)** | **0-pressure** |
| Patient with TA | Mean cardiac output:  6.11 l/min; HR: 60 | IA: 19.9%; LCCA: 6.7%;  LSCA: 3.4% | 0 Pa |
| Control | Mean cardiac output:  6.11 l/min; HR: 60 | IA: 16.4%; LCCA: 4.2%;  LSCA: 9.4% | 0 Pa |

AoR = Aortic arch branches; DAo = Descending aorta

As can be seen in Table S3, using a non-realistic BC (i.e. BC2 with 0-pressure being defined at the descending aorta (DA) outlet) failed to reproduce physiologically relevant pressure values, such that the mean pressure at the inlet is 18.6 and 3.9 mmHg for the TA and control model, respectively. However, BC2 could produce comparable velocity and TAWSS results in the control model, but this was not true for the TA model, in which the magnitudes of velocity and TAWSS were significantly increased, which might be caused by the greatly increased pressure drop from the inlet to the DA outlet.

**Table S3.** Comparison of results obtained from different sets of boundary conditions.

|  | **Patient with TA** | | | **Control** | | |
| --- | --- | --- | --- | --- | --- | --- |
|  | **Original BC** | **BC1** | **BC2** | **Original BC** | **BC1** | **BC2** |
| Max. Velocity (m/s) | 0.95 | 0.87 | 1.16 | 0.59 | 0.59 | 0.58 |
| Max. TAWSS (Pa) | 64.15 | 56.95 | 93.47 | 7.46 | 7.23 | 7.29 |
| Mean TAWSS (Pa) | 3.99 | 3.54 | 5.10 | 1.06 | 0.98 | 0.97 |
| Mean inlet pressure (mmHg) | 98.00 | 92.67 | 18.60 | 80.71 | 93.22 | 3.89 |
| Max. PD (mmHg) | 36.99 | 35.51 | 119.13 | 29.68 | 26.6 | 28.16 |

Regarding BC1, the control model produced similar results as compared to those obtained from the original BC, because the mean inlet cardiac outputs were comparable between the two BCs (Original BC: 6.15 l/min vs. BC 1: 6.11 l/min). Moreover, either the pressure values used to tune the 3-EMW (mean BP: Original BC: 83 mmHg vs. BC1: 93 mmHg), or the HR used to scale the inflow waveform seemed to have minor effects on the predicted results. In the TA model, the mean cardiac output with BC1 was reduced by approximately 8% as compared to the original BC, and this resulted in an obvious reduction in the magnitudes of velocity and TAWSS, by around 8% and 11%, respectively. Considering the BSA that were used to scale the mean cardiac output, are similar between the two groups (TA: 1.9 [1.8, 2.0] vs. 1.9 [1.7, 2.0] m2), the patient-specifically scaled flow waveforms applied at the models’ inlets would hardly affect the statistical results shown in the present study.

Moreover, despite assuming the same HR, BP, and inflow waveform for the TA and control models with BC1, the magnitudes of velocity, TAWSS, and PD obtained from the patient with TA are still obviously higher than those obtained from the control model, indicating the hemodynamic difference between the two groups might mainly come from different geometric features.

# **S4. Flow patterns**


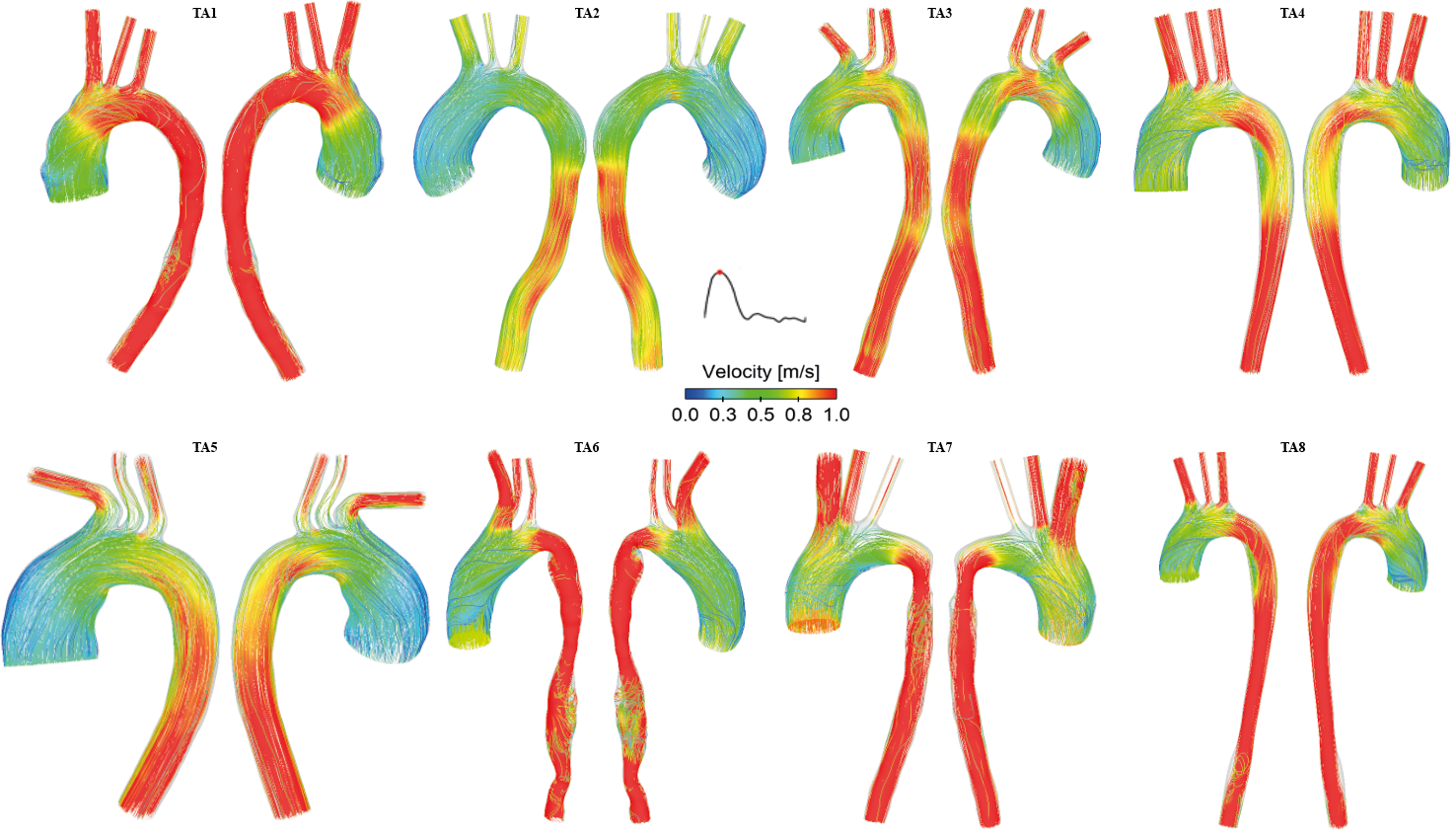


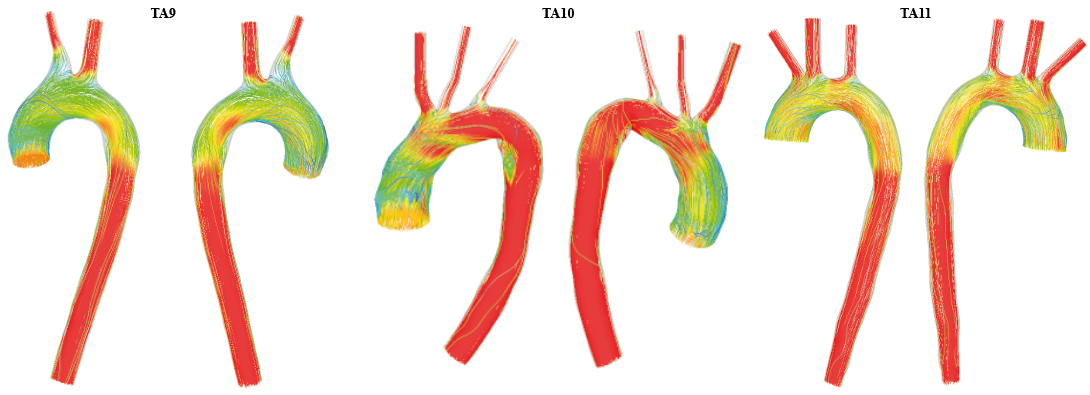


**Figure S2a**. Flow patterns for all patients with Takayasu arteritis (TA).


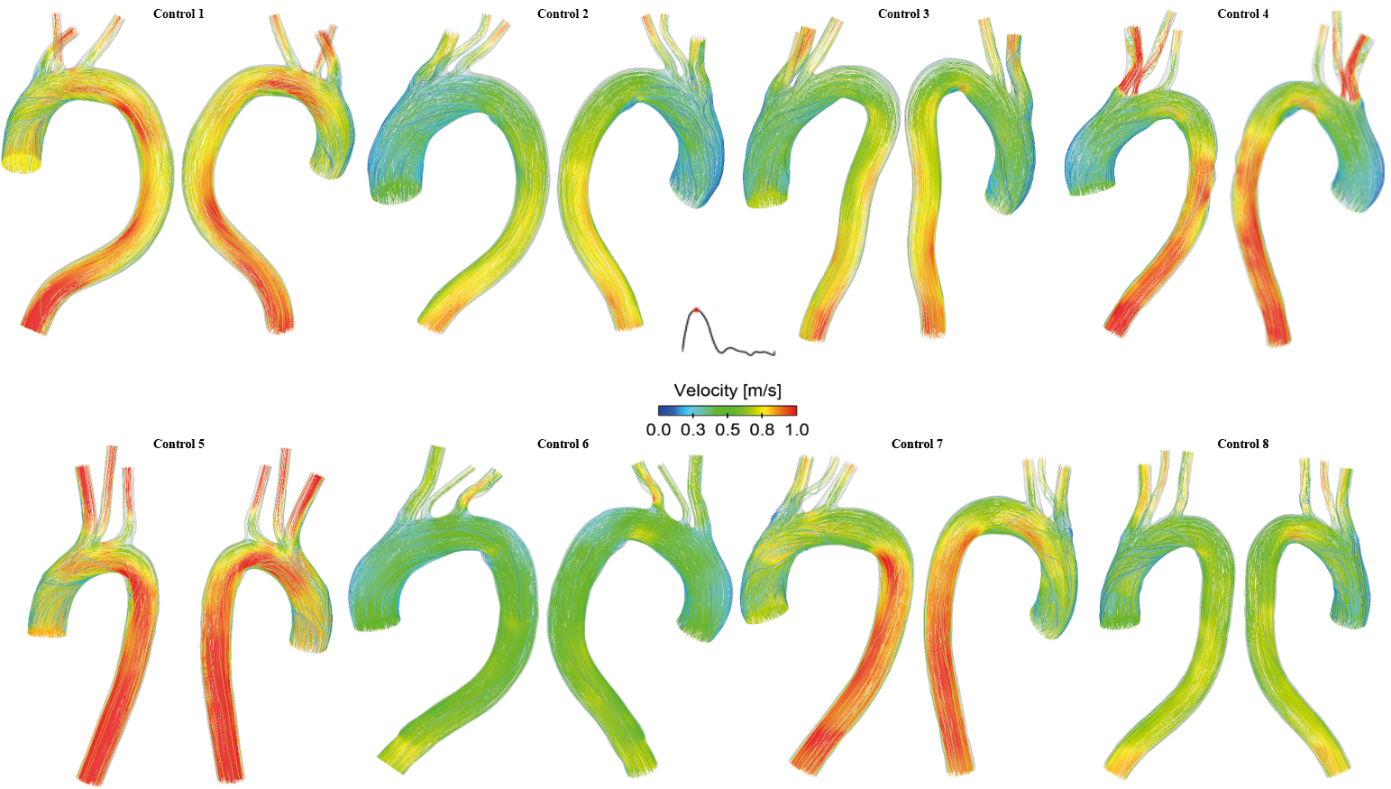

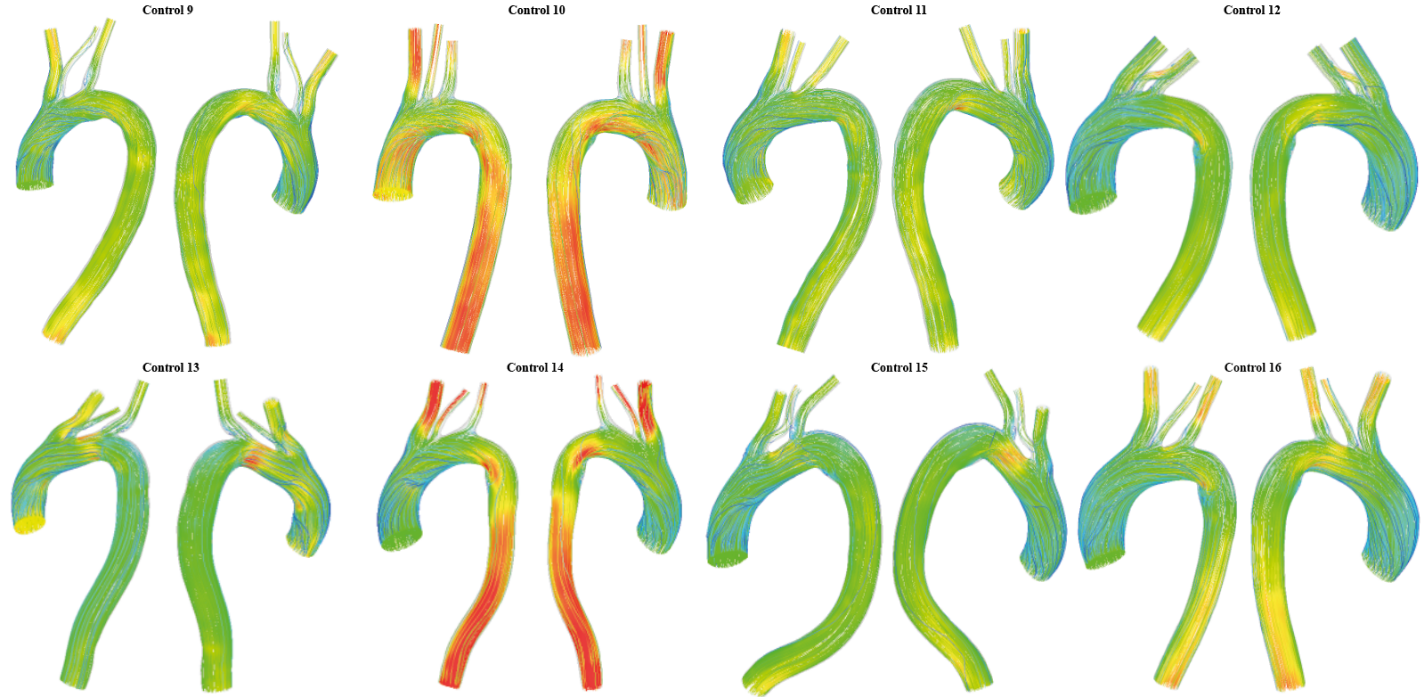

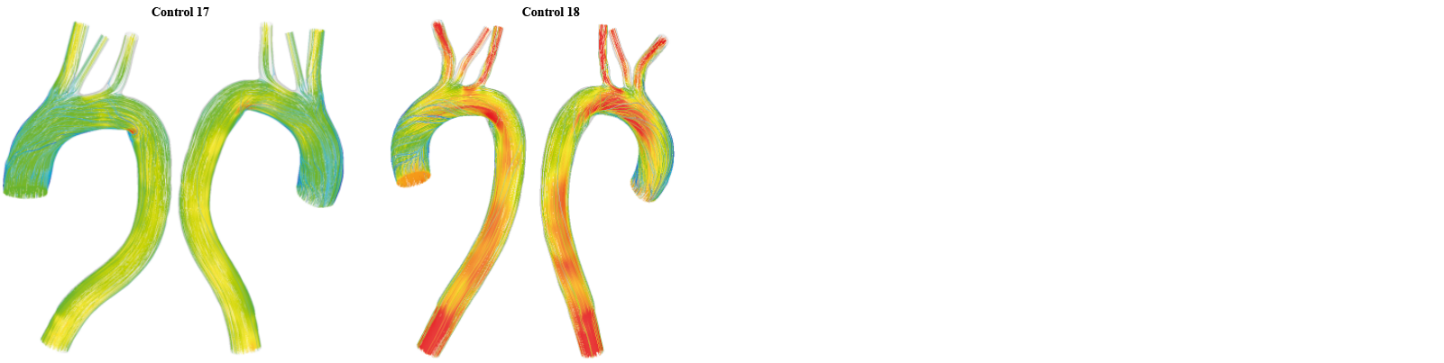


**Figure S2b**. Flow patterns for all patients in the control group.

# **S5. Time-averaged wall shear stress (TAWSS) distributions**


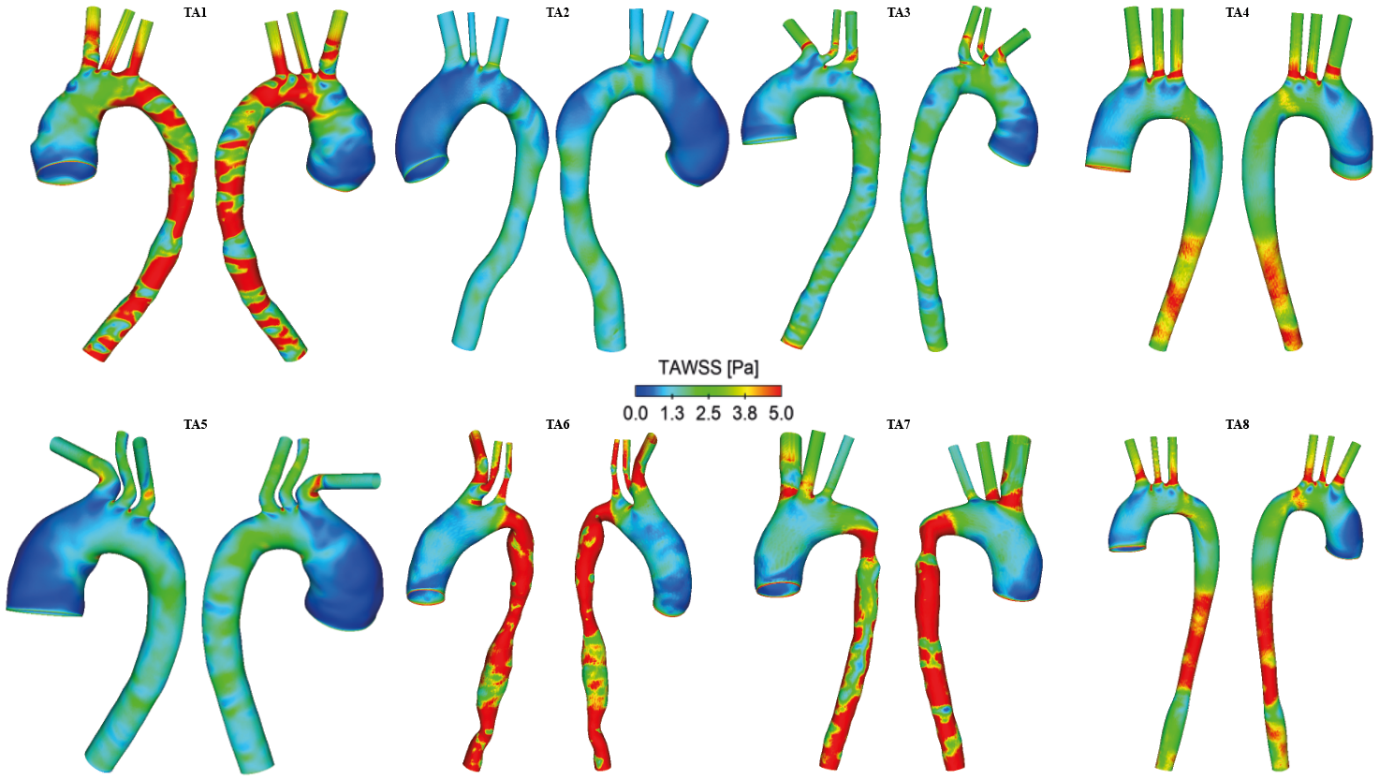

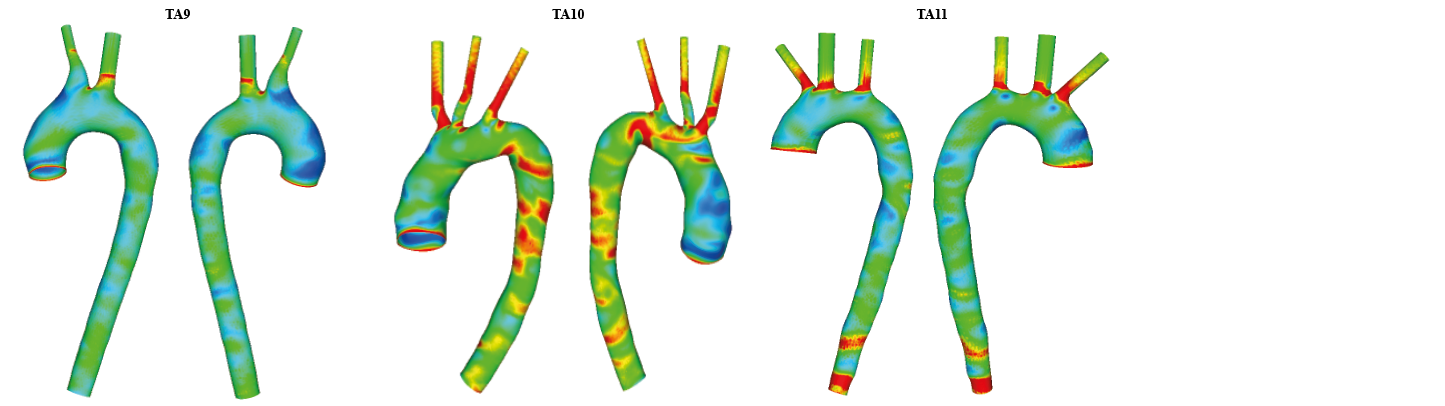


**Figure S3a**. TAWSS distributions for all patients with Takayasu arteritis (TA).


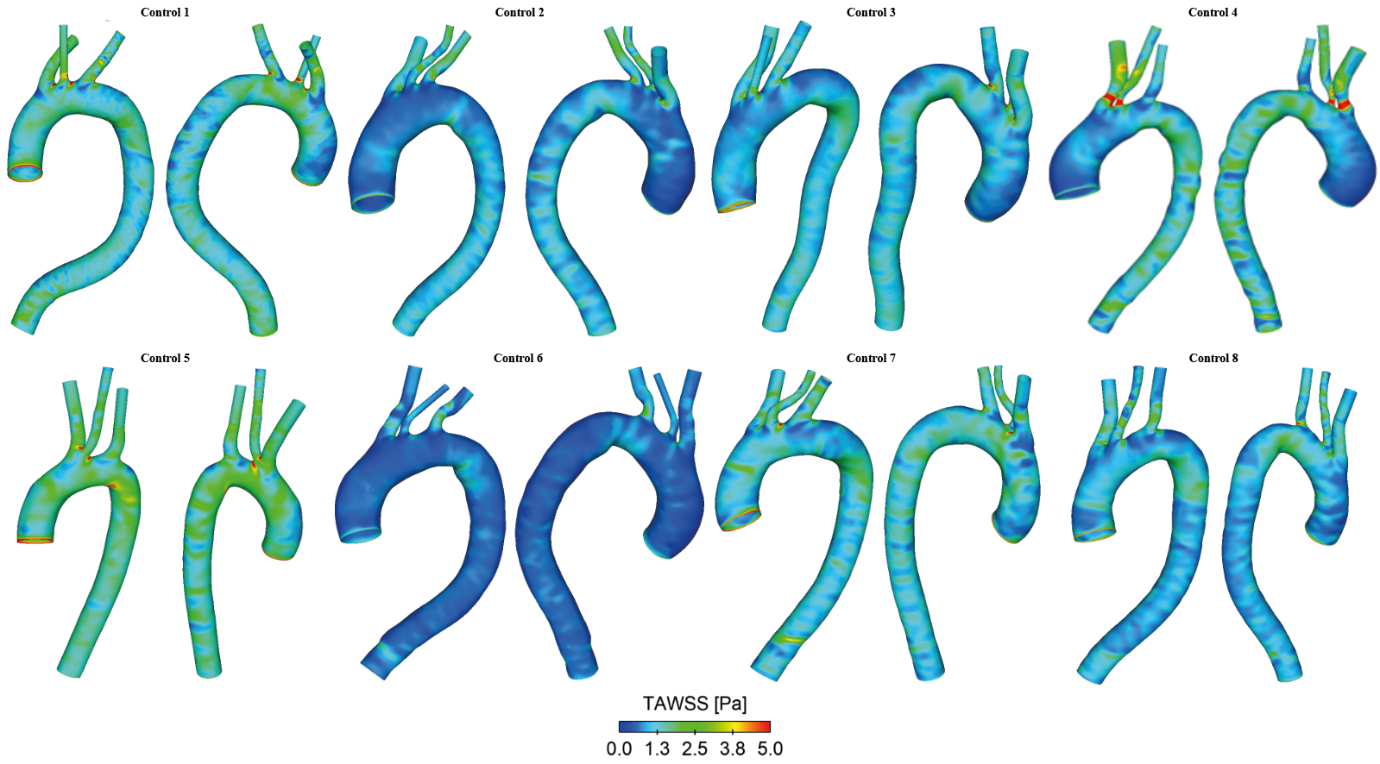

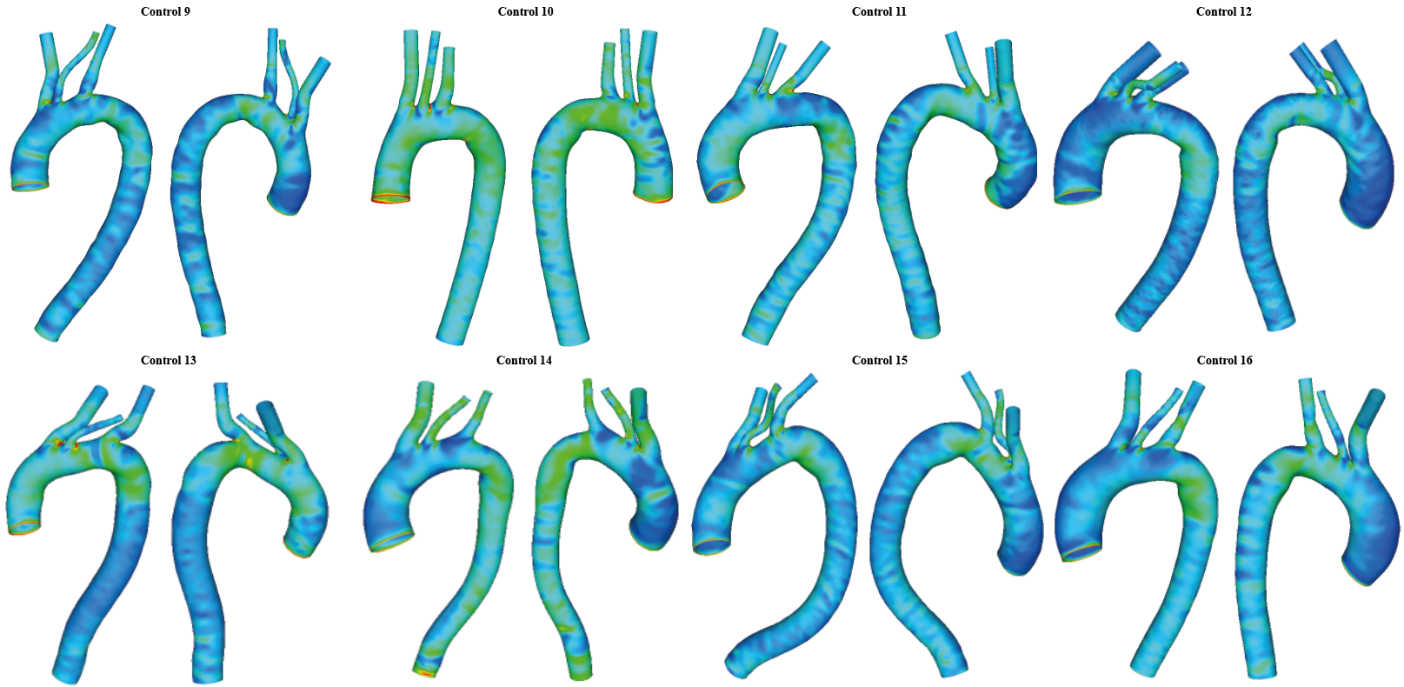

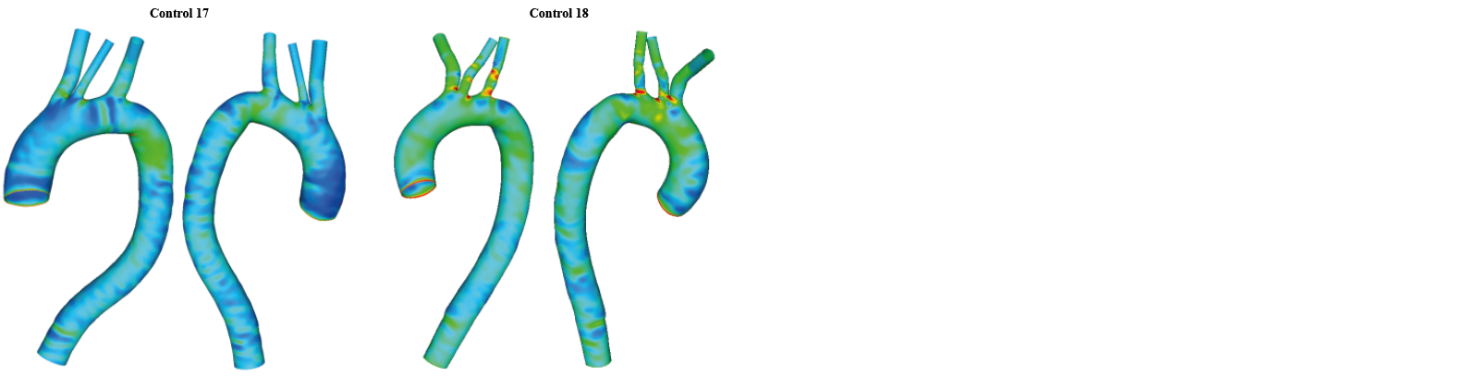


**Figure S3b**. TAWSS distributions for all patients in the control group.
